# Anticancer sulfonamide hybrids that inhibit bladder cancer cells growth and migration as tubulin polymerisation inhibitors

**DOI:** 10.1080/14756366.2019.1639696

**Published:** 2019-08-11

**Authors:** Jia Liu, Chunlai Liu, Xiling Zhang, Liu Yu, Xue Gong, Ping Wang

**Affiliations:** Department of Urology, The Fourth Affiliated Hospital of China Medical University, Shenyang, China

**Keywords:** Sulfonamide-dithiocarbamate, RT-112 cell, migration, tubulin polymerisation, antitumor

## Abstract

Novel sulfonamide-dithiocarbamate hybrids were designed and synthesised via the molecular hybridisation strategy. Among them, compound **13d** displayed a potent activity with IC_50_ values of 0.9, 0.7, 1.9 and 2.6 µM against UM-UC-3, RT-112, RT4 and T24. Compound **13d** inhibited the migration and regulated the migration-related markers (E-cadherin, N-cadherin, Vimentin, Snail and Slung) against RT-112 cells in a concentration dependent manner. By the tubulin polymerisation assay *in vitro* and immunostaining assay, compound **13d** was identified as a novel tubulin polymerisation inhibitor. Intragastric administration of compound **13d** could inhibit the growth of RT-112 cells *in vivo* in a xenograft mouse model with the low toxicity, indicating that it may be a leading candidate with antitumor properties to treat bladder cancer.

## Graphical Abstract

**Figure:**
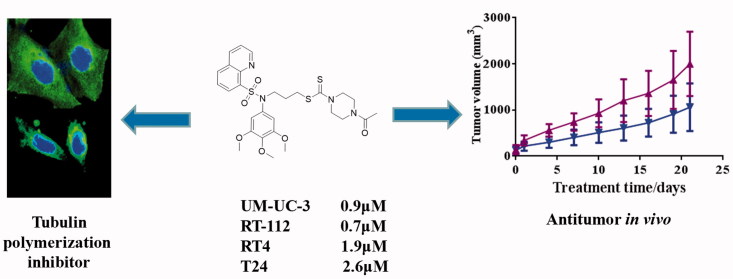

## Introduction

**Figure 1.**
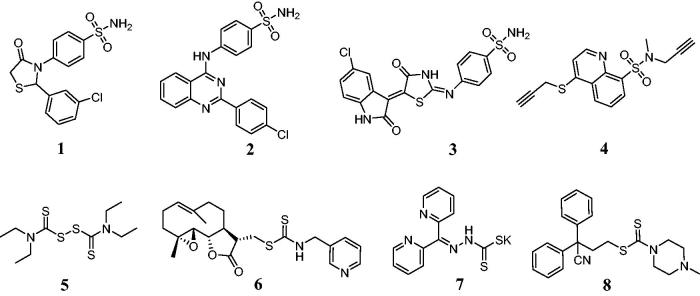
Antitumor sulfonamide and dithiocarbamate derivatives.

Sulfonamide derivatives were extensively studied due to a variety of biological activities such as antimicrobial, anticancer and antiviral properties^1–3^. Benzenesulfonamide derivative **1** (Figure 1) induced nuclear condensation, cell shrinkage, and nuclear fragmentation to apoptosis against COLO-205 cell line^4^. Sulfonamide derivative **2** could decrease the cell viability of HT-29 cell line in a concentration dependent manner and with an IC_50_ value of 5.45 µM^5^. Benzenesulfonamide **3** displayed a potent antiproliferative activity against MCF-7 cells with an IC_50_ value of 3.96 µM as an apoptosis inducer^6^. Quinolinesulfonamide **4** exhibited the anticancer activity *in vitro* against T47D cells with an IC_50_ value of 0.27 µM^7^. In addition, dithiocarbamates as organosulfur ligands were successfully applied as anticancer agents^8^. Disulfiram **5** could modulate ROS accumulation and overcome synergistically cisplatin resistance against breast cancer cells^9^. Dithiocarbamate **6** induced apoptosis by the mitogen-activated protein kinase signal pathway^10^. Dithiocarbamate **7** showed the potent anticancer effects against HER2-overexpressed SK-OV-3 and SK-BR-3 cells^11^. Dithiocarbamate **8** was developed as an antitumor candidate drug for the treatment of estrogen receptor-positive breast cancer^12^.

**Figure 2.**
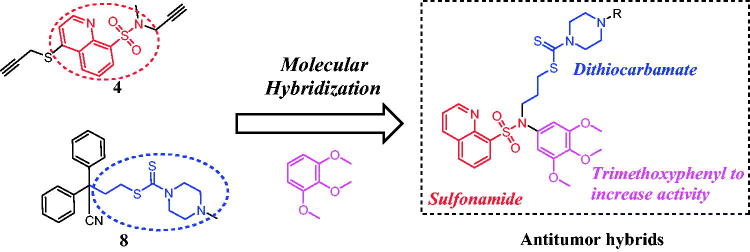
Illustration of the design strategy for sulfonamide hybrids.

Over the last decade, a variety of molecules bearing the trimethoxyphenyl moiety have been designed and synthesised as anticancer agents^13–15^. In addition, trimethoxyphenyl derivatives have enjoyed great success due to their potent chemical stability and pharmacokinetics for cancer treatment^16^. These above interesting findings led us to perform the molecular hybridisation strategy of biologically active sulfonamide, dithiocarbamate and trimethoxyphenyl fragments to generate a novel scaffold with the aim of studying the impact of such modification on their antitumor activity. As shown in Figure 2, a molecular hybridisation strategy based on bioactive compounds **4** and **8** produced a novel scaffold that has three parts: (1) a sulfonamide fragment as the central backbone; (2) a dithiocarbamate moiety and (3) a trimethoxyphenyl moiety to increase the antiproliferative activity. In this work, we synthesised and evaluated for the anticancer mechanisms *in vitro* and *in vivo* of sulfonamide-dithiocarbamate hybrids.

## Methods and materials

### Reagents and chemicals

All the chemical reagents were purchased from Sigma Aldrich (Shanghai, China), Aladdin (Shanghai, China), and Ruida company (Shandong, China). ^1^H NMR (400 MHz) and ^13^C NMR (100 MHz) spectra were measured using CDCl_3_ and DMSO-*d_6_* as solvents. High-resolution mass spectra (HRMS) spectrometry (Ruida company, China) was performed in this work.

### Synthesis of compound 11

A mixture of quinoline-8-sulfonyl chloride **9** (5 mmol), 3,4,5-trimethoxyaniline **10** (5 mmol), and NaHCO_3_ (5 mmol) were dissolved in CH_2_Cl_2_ (15 ml). The mixture was stirred for 4 h at room temperature. The crude product was purified by column chromatography (*n*-hexane:ethyl acetate = 6:1) on silica gel to afford compound **11.**

### Synthesis of compound 12

Compound **11** (3 mmol), 1,3-dibromopropane (4.5 mmol) and NaOH (3 mmol) was dissolved in acetone (15 ml). The mixture was refluxed for 8 h. The crude product was purified by column chromatography (*n*-hexane:ethyl acetate = 9:1) on silica gel to afford compound **12.**

### Synthesis of sulfonamide hybrids 13a–13d

Compound **12** (2 mmol), triethylamine (3 mmol) and piperazine derivatives (2 mmol) were dissolved in MeOH (10 ml). CS_2_ (4 mmol) was added to this reaction mixture at 0 °C. The reaction mixture was stirred for 1 h at 0 °C. The crude product was purified by column chromatography (*n*-hexane:ethyl acetate = 8:1) on silica gel to afford hybrids **13a**∼**13d.**

### N-(3,4,5-trimethoxyphenyl)quinoline-8-sulfonamide (11)

White solid, yield: 94%, m.p.: 1 8 1 ∼ 183 °C. ^1^H NMR (400 MHz, DMSO-*d_6_*) δ 9.87 (s, 1H), 9.16 (dd, *J* = 4.2, 1.7 Hz, 1H), 8.52 (dd, *J* = 8.4, 1.7 Hz, 1H), 8.39 (dd, *J* = 7.3, 1.3 Hz, 1H), 8.27 (dd, *J* = 8.2, 1.3 Hz, 1H), 7.81 – 7.60 (m, 2H), 6.35 (s, 2H), 3.51 (s, 6H), 3.47 (s, 3H). ^13^C NMR (100 MHz, DMSO-*d_6_*) δ 152.6, 151.4, 142.7, 136.0, 135.1, 134.2, 133.9, 133.6, 132.3, 128.3, 125.6, 122.6, 98.0, 59.9, 55.6. HRMS (m/z): Calcd. C_18_H_19_N_2_O_5_S, [M + H]^+^m/z: 375.1015, found: 375.1018.

### N-(3-bromopropyl)-N-(3,4,5-trimethoxyphenyl)quinoline-8-sulfonamide (12)

White solid, yield: 82%, m.p.: 1 2 9 ∼ 131 °C. ^1^H NMR (400 MHz, CDCl_3_) δ 9.09 (dd, *J* = 4.2, 1.7 Hz, 1H), 8.20 (td, *J* = 8.6, 1.5 Hz, 2H), 7.93 (dd, *J* = 8.2, 1.2 Hz, 1H), 7.55 – 7.39 (m, 2H), 6.12 (s, 2H), 4.22 (t, *J* = 6.8 Hz, 2H), 3.67 (d, *J* = 4.6 Hz, 3H), 3.47 (t, *J* = 6.8 Hz, 2H), 3.42 (d, *J* = 8.3 Hz, 6H), 2.09 (p, *J* = 6.8 Hz, 2H). ^13 ^C NMR (100 MHz, CDCl_3_) δ 152.0, 150.1, 143.2, 136.6, 135.8, 135.6, 133.6, 133.1, 132.5, 127.7, 124.6, 121.0, 105.2, 59.8, 54.9, 51.0, 31.8, 29.5. HRMS (m/z): Calcd. C_21_H_24_BrN_2_O_5_S, [M + H]^+^m/z: 495.0589, found: 495.0593.

### 3-(N-(3,4,5-trimethoxyphenyl)quinoline-8-sulfonamido)propyl-4-methylpiperazine-1-carbodithioate (13a)

White solid, yield: 76%, m.p.: 9 6 ∼ 98 °C. ^1^H NMR (400 MHz, CDCl_3_) δ 9.10 (dd, *J* = 4.2, 1.7 Hz, 1H), 8.37–8.08 (m, 2H), 7.93 (dd, *J* = 8.2, 1.2 Hz, 1H), 7.64–7.29 (m, 2H), 6.13 (s, 2H), 4.32 (s, 2H), 4.20 (t, *J* = 6.8 Hz, 2H), 3.96 (s, 2H), 3.67 (s, 3H), 3.42 (s, 6H), 3.40 (d, *J* = 7.3 Hz, 2H), 2.50 (s, 4H), 2.30 (s, 3H), 1.99–1.82 (m, 2H). ^13 ^C NMR (100 MHz, CDCl_3_) δ 151.9, 150.2, 143.2, 136.5, 136.0, 135.5, 133.6, 133.1, 132.3, 127.7, 124.6, 121.0, 105.4, 59.8, 54.9, 53.2, 51.5, 44.3, 33.3, 27.8. HRMS (m/z): Calcd. C_27_H_35_N_4_O_5_S_3_, [M + H]^+^m/z: 591.1770, found: 591.1776.

### 3-(N-(3,4,5-trimethoxyphenyl)quinoline-8-sulfonamido)propyl-4-ethylpiperazine-1-carbodithioate(13b)

White solid, yield: 84%, m.p.: 1 3 1 ∼ 133 °C. ^1^H NMR (400 MHz, CDCl_3_) δ 9.21–8.97 (m, 1H), 8.20 (dd, *J* = 9.8, 8.1 Hz, 2H), 7.92 (d, *J* = 7.7 Hz, 1H), 7.72–7.32 (m, 2H), 6.14 (s, 2H), 4.24 (d, *J* = 26.0 Hz, 2H), 4.20 (t, *J* = 6.7 Hz, 2H), 3.89 (s, 2H), 3.67 (s, 3H), 3.43 (s, 6H), 3.39 (d, *J* = 7.3 Hz, 2H), 2.64–2.17 (m, 6H), 1.99–1.83 (m, 2H), 1.03 (t, *J* = 7.2 Hz, 3H). ^13^C NMR (100 MHz, CDCl_3_) δ 195.8, 151.9, 150.2, 143.3, 136.5, 136.0, 135.5, 133.6, 133.0, 132.3, 127.7, 124.6, 121.0, 105.5, 59.8, 54.9, 51.6, 51.2, 50.9, 33.1, 27.8, 11.0. HRMS (m/z): Calcd. C_28_H_37_N_4_O_5_S_3_, [M + H]^+^m/z: 605.1926, found: 605.1929.

### 3-(N-(3,4,5-trimethoxyphenyl)quinoline-8-sulfonamido)propyl-4–(2-hydroxyethyl)piperazine-1-carbodithioate(13c)

White solid, yield: 75%, m.p.: 1 0 6 ∼ 108 °C. ^1^H NMR (400 MHz, CDCl_3_) δ 9.10 (d, *J* = 2.1 Hz, 1H), 8.20 (dd, *J* = 6.8, 3.9 Hz, 2H), 7.93 (d, *J* = 8.1 Hz, 1H), 7.65–7.34 (m, 2H), 6.12 (s, 2H), 4.33 (s, 2H), 4.20 (t, *J* = 6.6 Hz, 2H), 3.96 (s, 2H), 3.67 (s, 3H), 3.66–3.57 (m, 2H), 3.42 (s, 8H), 2.73–2.49 (m, 6H), 1.93 (dd, *J* = 13.7, 6.8 Hz, 2H). ^13^C NMR (100 MHz, CDCl_3_) δ 196.3, 151.9, 150.2, 143.2, 136.3, 135.9, 135.5, 133.5, 133.1, 132.4, 127.6, 124.6, 121.0, 105.3, 59.8, 58.2, 56.7, 54.9, 51.5, 51.3, 33.2, 27.7. HRMS (m/z): Calcd. C_28_H_37_N_4_O_6_S_3_, [M + H]^+^m/z: 621.1875, found: 621.1878.

### 3-(N-(3,4,5-trimethoxyphenyl)quinoline-8-sulfonamido)propyl-4-acetylpiperazine-1-carbodithioate (13d)

White solid, yield: 90%, m.p.: 1 2 8 ∼ 129 °C. ^1^H NMR (400 MHz, CDCl_3_) δ 9.09 (dd, *J* = 4.2, 1.7 Hz, 1H), 8.27–8.09 (m, 2H), 7.93 (dd, *J* = 8.2, 1.1 Hz, 1H), 7.56–7.33 (m, 2H), 6.13 (s, 2H), 4.21 (t, *J* = 6.7 Hz, 2H), 4.19 (s, 4H), 3.66 (s, 3H), 3.65 (s, 2H), 3.55–3.49 (m, 2H), 3.45 (s, 2H), 3.42 (s, 6H), 2.06 (s, 3H), 1.94 (dd, *J* = 14.9, 7.7 Hz, 2H). ^13^C NMR (100 MHz, CDCl_3_) δ 197.0, 168.4, 152.0, 150.2, 143.2, 136.5, 135.9, 135.6, 133.5, 133.1, 132.4, 127.6, 124.6, 121.0, 105.4, 59.8, 54.9, 51.6, 44.2, 39.6, 33.2, 27.7, 20.4. HRMS (m/z): Calcd. C_28_H_35_N_4_O_6_S_3_, [M + H]^+^m/z: 619.1719, found: 619.1726.

### Cell proliferation

Breast cancer cells (MDA-MB-453), liver cancer cells (SNU-423), and bladder cancer cells (UM-UC-3, RT-112, RT4, T24) were purchased from the Shanghai culture company, and cultured at 37 °C in an atmosphere containing 5% CO_2_. 4,000 cells per well were seeded into a 96-well plate for 24 h and compounds were added to cluture 48 h. Next, 6 mg/ml CCK8 (20 µl) per well was added to explore the cell survival.

### Migration assay

Transwell chamber was used to do migration assay according to the reported references^17^^,^^18^. Ten thousand RT-112 cells containing compound **13d** were seeded in the top chambers for 24 h incubation. The bottom chambers were fixed and stained with crystal violet for 2 h. Finally, the chambers were washed with water, and the top membrane was left to dry.

### Western blot

RT-112 cells were treated with compound **13d** and then lysed in a RIPA lysis buffer. The protein samples of RT-112 cells were electrophoresed on SDS-PAGE. Nextly, targeted proteins were transferred to PVDF membrane and incubated with antibodies (E-cadherin, N-cadherin, Vimentin, Snail and Slung). The markers were visualised by chemiluminescence detection reagents.

### Tubulin polymerisation assay and immunostaining

An amount of 6 mg/ml tubulin was resuspended in the PEM buffer (80 mM PIPES (pH 6.9), 1 mM EGTA, 0.5 mM MgCl_2_, 1 mM ATP, 10.2% (v/v) glycerol) and then was preincubated with a sulfonamide-dithiocarbamate hybrid **13d** on the ice. The reaction was monitored by a spectrophotometer (Beijng Tianhe Company) at 37 °C to obtain the results of tubulin polymerisation *in vitro*. RT-112 cells were treated with sulfonamide-dithiocarbamate hybrid **13d** for 48 h. Then, slices were fixed by 4% paraformaldehyde for 20 min and washed by PBS for five times. 0.6% Triton-X-100 was added and 0.1% BSA was used to block. β-tubulin antibody was added to incubate overnight at 4 °C. DAPI was used to stain and then images were captured.

### The in vivo antitumor effect of compound 13d

*In vivo* experiments were processed according to guidelines established by the ethics committee of the 4th affiliated hospital of CMU. Mice were implanted with RT-112 cells (1.5 × 10^7^ cells per mouse) on the right flank of nude mice. The mice were divided into the saline group and sulfonamide-dithiocarbamate hybrid **13d** (80 mg/kg) group (*n* = 5 mice for each group). Intragastric administration of hybrid **13d** was performed every day for a period of 21 days.

## Results and discussion

### Synthesis of novel sulfonamide-dithiocarbamate hybrids

**Scheme 1.**
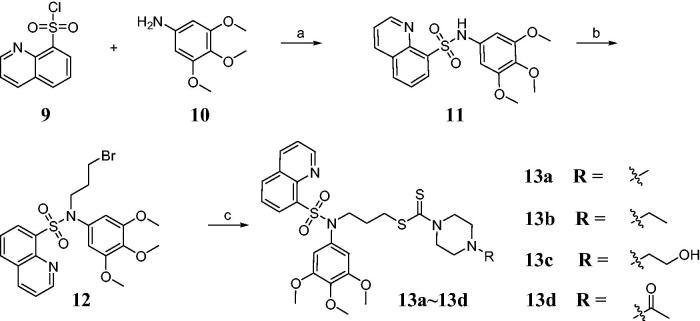
Reagents and conditions: (a) NaHCO_3_, CH_2_Cl_2_, rt; (b) 1,3-Dibromopropane, NaOH, acetone, reflux and (c) CS_2_, triethylamine, piperazine derivatives, MeOH, 0 °C.

The sulfonamide-dithiocarbamate hybrids **13a**–**13d** were synthesised according to Scheme 1. The desired compound **11** was obtained by the sulfonylation reaction of quinoline-8-sulfonyl chloride **9** and 3,4,5-trimethoxyaniline **10**. For synthesis of compound **12**, 1,3-dibromopropane was used in the presence of sodium hydroxide. Subsequently, intermediate **12** was treated with carbon disulfide and piperazine derivatives in the methanol solution to give sulfonamide-dithiocarbamate hybrids **13a**–**13d**. The chemical structures of obtained compounds were confirmed by ^1^H NMR, ^13^C NMR and mass spectroscopic data which were consistent with the proposed molecular structures.

### Anticancer activity in vitro of sulfonamide hybrids 13a–13d

CCK8 method was used to investigate the anticancer activity *in vitro* of sulfonamide-dithiocarbamate hybrids **13a**–**13d** against cancer cells for 48 h. MDA-MB-453 (breast cancer cells), SNU-423 (liver cancer cells) and RT-112 (bladder cancer cells) were selected to explore the antiproliferative activity. In this work, 5-fluorouracil (5-Fu) was used as the control drug. The results of anticancer activity *in vitro* were listed in Table 1.

**Table 1.** Anticancer activity *in vitro* of sulfonamide hybrids **13a–13d**.

Compound		IC_50_ (µM)^a^	
	MCF-7	SNU-423	RT-112
**11**	>40	>40	>40
**12**	>40	>40	>40
**13a**	6.4 ± 1.2	8.7 ± 0.9	7.8 ± 0.4
**13b**	8.5 ± 0.7	5.4 ± 0.3	6.8 ± 0.7
**13c**	4.2 ± 0.1	3.6 ± 0.7	4.1 ± 0.6
**13d**	2.8 ± 0.2	3.0 ± 0.1	0.7 ± 0.1
**5-Fu**	7.4 ± 0.5	10.8 ± 0.7	13.6 ± 0.2

a CCK8 assay after treatment for 48 h. The data are presented as the means of three independent experiments.

Compound **11** and compound **12** displayed the weak antiproliferative activity against all three cancer cells with IC_50_ values of >40 µM. However, sulfonamide hybrids **13a**–**13d** containing a dithiocarbamate moiety showed the potent activity with IC_50_ values from 0.7 to 8.7 µM. All these inhibitory results exhibited that the dithiocarbamate scaffold might play the pivotal effect for the anticancer activity *in vitro* of sulfonamide hybrids.

In addition, the effect of different piperazine units were explored. Replacement of the 1-methylpiperazine of compound **13a** with a 1-ethylpiperazine of compound **13b** led to an increase for the anticancer activity *in vitro* against SNU-423 and RT-112 cells. However, changing the 2-(piperazin-1-yl)ethan-1-ol of compound **13c** to a 1-ethylpiperazine of compound **13b** led to a decrease for the anticancer activity *in vitro* against all three cell lines. Importantly, sulfonamide hybrid **13d** exhibited the best antiproliferative activity with an IC_50_ value of 0.7 µM against bladder cancer RT-112 cells among all sulfonamide-dithiocarbamate analogs.

### Compound 13d inhibited the cell proliferation against bladder cancer cells

**Figure 3.**
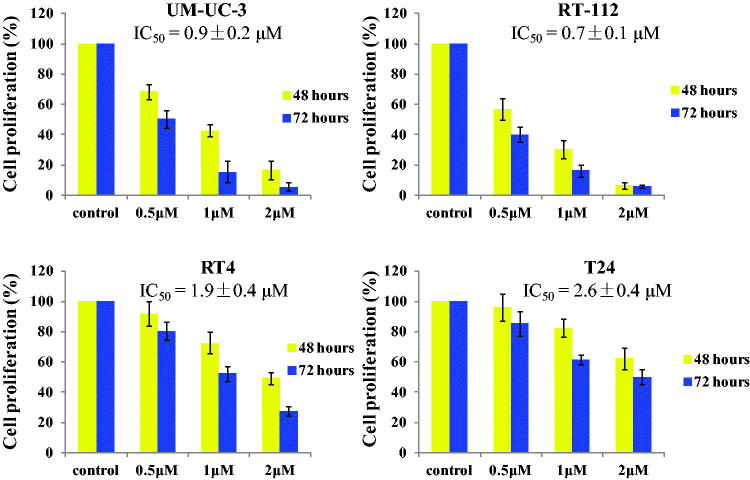
Cell proliferation of bladder cancer with the treatment of **13d**.

In order to explore the antiproliferation ability, four bladder cancer cell lines (UM-UC-3, RT-112, RT4 and T24) were selected and treated with the hybrid **13d** in the 24-well plate for 48 h and 72 h. As shown in Figure 3, compound **13d** inhibited the cell proliferation against bladder cancer in a concentration dependent manner. After the treatment for 48 h, compound **13d** displayed the potent activity with IC_50_ values of 0.9, 0.7, 1.9 and 2.6 µM against UM-UC-3, RT-112, RT4 and T24 cells. Compared with the cell proliferation of 48 h, the cell proliferation of 72 h was decreased. All these results indicated that compound **13d** could inhibit the cell proliferation against bladder cancer cells in concentration-dependent and time-dependent manners.

### Compound 13d inhibited the migration against RT-112 cells

**Figure 4.**
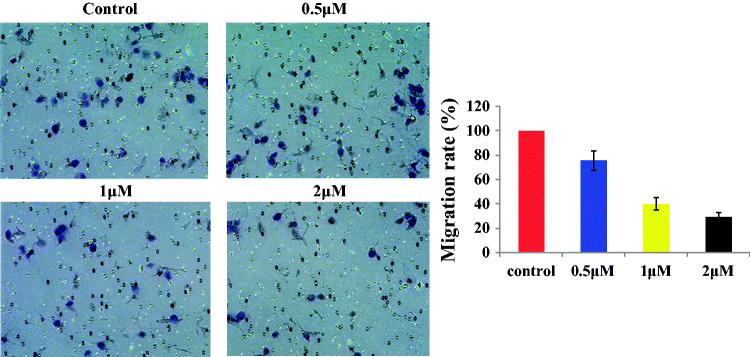
Migration rate of RT-112 cells with the treatment of **13d** for 48 h.

Based on the antiproliferative activity results, compound **13d** was selected to investigate the anticancer mechanisms against the RT-112 cell line. Corning transwell plate was used to do migration assay against RT-112 cells for 48 h. With the treatment of sulfonamide-dithiocarbamate hybrid **13d** at 0.5, 1 and 2 µM, the migration rate of RT-112 cells was 76%, 40% and 29%, respectively. The migration results in Figure 4 showed that compound **13d** inhibited the migration against RT-112 cells in a concentration dependent manner.

### Compound 13d regulated the migration-related markers against RT-112 cells

**Figure 5.**
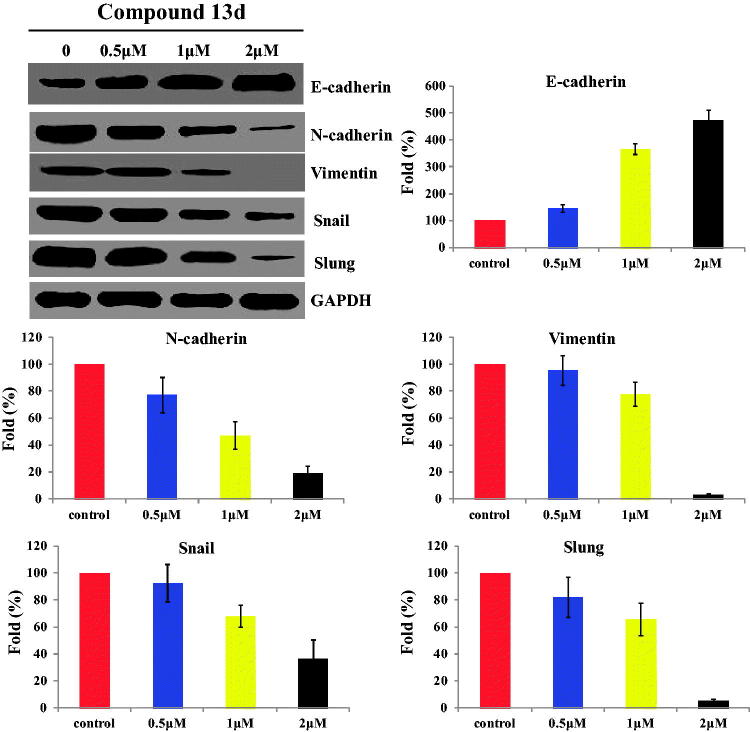
Compound **13d** regulated the migration-related proteins (E-cadherin, N-cadherin, Vimentin, Snail and Slung) against RT-112 cells.

To further characterise the migration effect of sulfonamide-dithiocarbamate hybrid **13d**, the expression levels of migration-related proteins (E-cadherin, N-cadherin, Vimentin, Snail and Slung) were investigated against RT-112 cells. Compound **13d** was treated for 48 h at concentrations of 0.5, 1 and 2 µM, respectively. As shown in Figure 5, sulfonamide-dithiocarbamate hybrid **13d** decreased the expression of N-cadherin, Vimentin, Snail and Slung in a concentration manner. In addition, hybrid **13d** increased the expression of E-cadherin in a concentration manner. All western blotting results revealed that compound **13d** could regulate the migration-related markers against RT-112 cells.

### Tubulin polymerisation inhibitory activity in vitro of compound 13d

**Figure 6.**
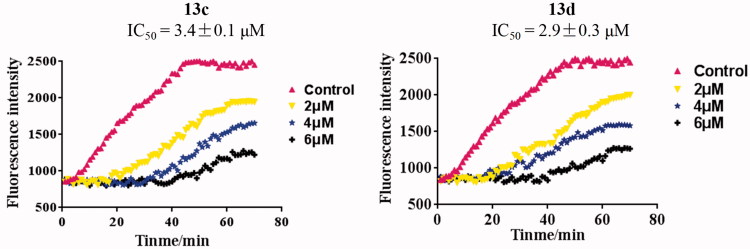
Tubulin polymerisation inhibitory activity *in vitro* of compounds **13c** and **13d**.

Because of the trimethoxyphenyl derivatives used as tubulin polymerisation inhibitors^19^, the most potent hybrids **13c** and **13d** containing the trimethoxyphenyl ring were evaluated for their tubulin polymerisation inhibitory activity *in vitro*. As shown in Figure 6, the time-course analisis of tubulin polymerisation at the concentrations (control, 2, 4 and 6 µM) of **13c** and **13d**. The weak inhibition was observed at 2 µM and hybrids **13c** and **13d** significantly inhibited tubulin polymerisation at 6 µM. The IC_50_ values of hybrids **13c** and **13d** for tubulin polymerisation inhibitory activity *in vitro* were 3.4 and 2.9 µM, respectively. Tubulin polymerisation assay investigated that compound **13d** inhibited tubulin polymerisation in a concentration dependent manner.

### Compound 13d inhibited tubulin polymerisation by the immunostaining assay

**Figure 7.**
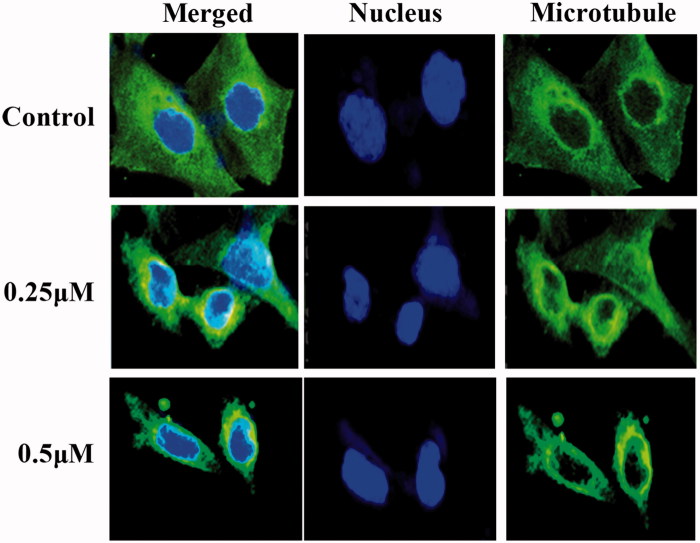
Tubulin polymerisation inhibitory effects of **13d** by the immunostaining assay.

Tubulin polymerisation inhibitory effects of the sulfonamide-dithiocarbamate hybrid **13d** were further explored by the immunofluorescent staining in RT-112 cells. From the results in Figure 7, the microtubule networks in RT-112 cells without the treatment of **13d** had a normal arrangement with microtubules extending from the central regions of the cell to the cell periphery. However, after the exposure to 0.25 µM of compound **13d**, the microtubule organisation in the cytosol were disrupted. The changes of the mitotic spindles were more clearly observed by increasing the concentration to 0.5 µM. All these results by the immunostaining assay indicated that compound **13d** disrupted the microtubule organisation of RT-112 cells at low concentrations.

### Compound 13d inhibited the tumor growth of RT-112 cells

**Figure 8.**
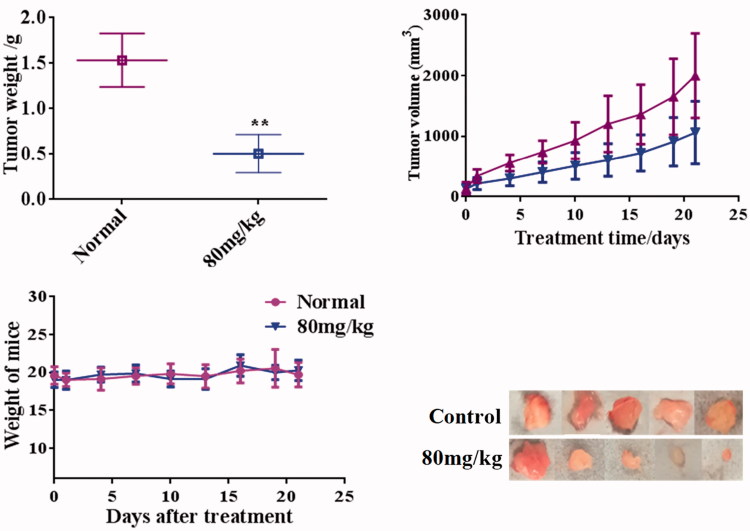
Compound **13d** inhibited the tumor growth of RT-112 cells.

In order to investigate the tumor growth effects of compound **13d**, the xenograft nude mouse of RT-112 cells were established. From the antitumor results in Figure 8, there is no significant difference in body weights between control and **13d** treated groups (80 mg/kg). The tumor volume of **13d** treated mice was obviously less than that of the control group. The average tumor weights of control and 80 mg/kg **13d** groups were 1.53 ± 0.30 g and 0.51 ± 0.21 g (inhibitory rate: 66.67%). These results showed that compound **13d** inhibited the tumor growth of RT-112 cells with the low toxicity.

## Conclusion

Bladder cancer is one of the most costly cancers to treat due to the considerable costs associated with the life-long clinical management and bladder tumors are not easy to cure because of their high metastasis rates. Therefore, it is very necessary to develop novel antitumor agants to treat bladder cancer. In this work, we designed novel sulfonamide-dithiocarbamate hybrids by the molecular hybridisation strategy based on reported bioactive scaffolds. Among all sulfonamide-dithiocarbamate hybrids, compound **13d** displayed the potent activity with IC_50_ values of 0.9, 0.7, 1.9 and 2.6 µM against UM-UC-3, RT-112, RT4 and T24 cells. In addition, **13d** inhibited the cell proliferation against bladder cancer cell lines by concentration-dependent and time-dependent manners.

From the migration assay, we found that compound **13d** inhibited the migration against RT-112 cells in a concentration dependent manner. With the treatment of compound **13d** at 2 µM, the migration rate of RT-112 cells was obviously decreased to 29% compared with control groups. By the western blotting experiments, **13d** decreased the expression of N-cadherin, Vimentin, Snail and Slung and increased the expression of E-cadherin in a concentration manner.

Furthermore, the microtubule organisation in the cytosol of RT-112 cells was disrupted with the treatment of compound **13d**. The IC_50_ value of hybrid **13d** for tubulin polymerisation inhibitory activity *in vitro* was 2.9 µM. By the tubulin polymerisation *in vitro* assay and immunofluorescent staining experiments, compound **13d** was identified as a novel tubulin polymerisation inhibitor. More importantly, compound **13d** inhibited the tumor growth of RT-112 cells *in vivo* with the low toxicity. In summary, compound **13d** may be a leading candidate with antitumor properties to treat bladder cancer.

## Supplementary Material

Supplemental Material
