# Supplementary material for: Anticancer sulfonamide hybrids that inhibit bladder cancer cells growth and migration as tubulin polymerisation inhibitors
Source: J Enzyme Inhib Med Chem. 2019 Aug 11;34(1):1380–7. doi: 10.1080/14756366.2019.1639696 (PMC6713100; doi:10.1080/14756366.2019.1639696)
Supplement: Supplemental Material [file IENZ_A_1639696_SM9168.pdf]

# Anticancer sulfonamide hybrids that inhibit bladder cancer cells growth and migration as tubulin polymerization inhibitors

Jia Liu[a], Chunlai Liu[a], Xiling Zhang[a], Liu Yu[a], Xue Gong[a], Ping Wang[*a]
[a]Department of Urology, The Fourth Affiliated Hospital of China Medical University, Shenyang, 110032, China.
[*]Corresponding author: Ping Wang (drpingwang@yeah.net)

***N-(3,4,5-trimethoxyphenyl)quinoline-8-sulfonamide (11)***

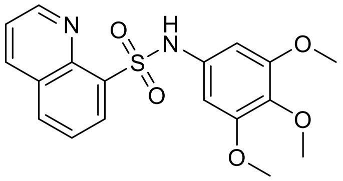


White solid, yield:94%, m.p.:181~183 °C. $^{1}$H NMR (400 MHz, DMSO-$d_6$) δ 9.87 (s, 1H), 9.16 (dd, $J$ = 4.2, 1.7 Hz, 1H), 8.52 (dd, $J$ = 8.4, 1.7 Hz, 1H), 8.39 (dd, $J$ = 7.3, 1.3 Hz, 1H), 8.27 (dd, $J$ = 8.2, 1.3 Hz, 1H), 7.81 – 7.60 (m, 2H), 6.35 (s, 2H), 3.51 (s, 6H), 3.47 (s, 3H). $^{13}$C NMR (100 MHz, DMSO-$d_6$) δ 152.6, 151.4, 142.7, 136.0, 135.1, 134.2, 133.9, 133.6, 132.3, 128.3, 125.6, 122.6, 98.0, 59.9, 55.6. HRMS (m/z): Calcd. $C_{18}H_{19}N_2O_5S$, $[M+H]^+$m/z: 375.1015, found: 375.1018.

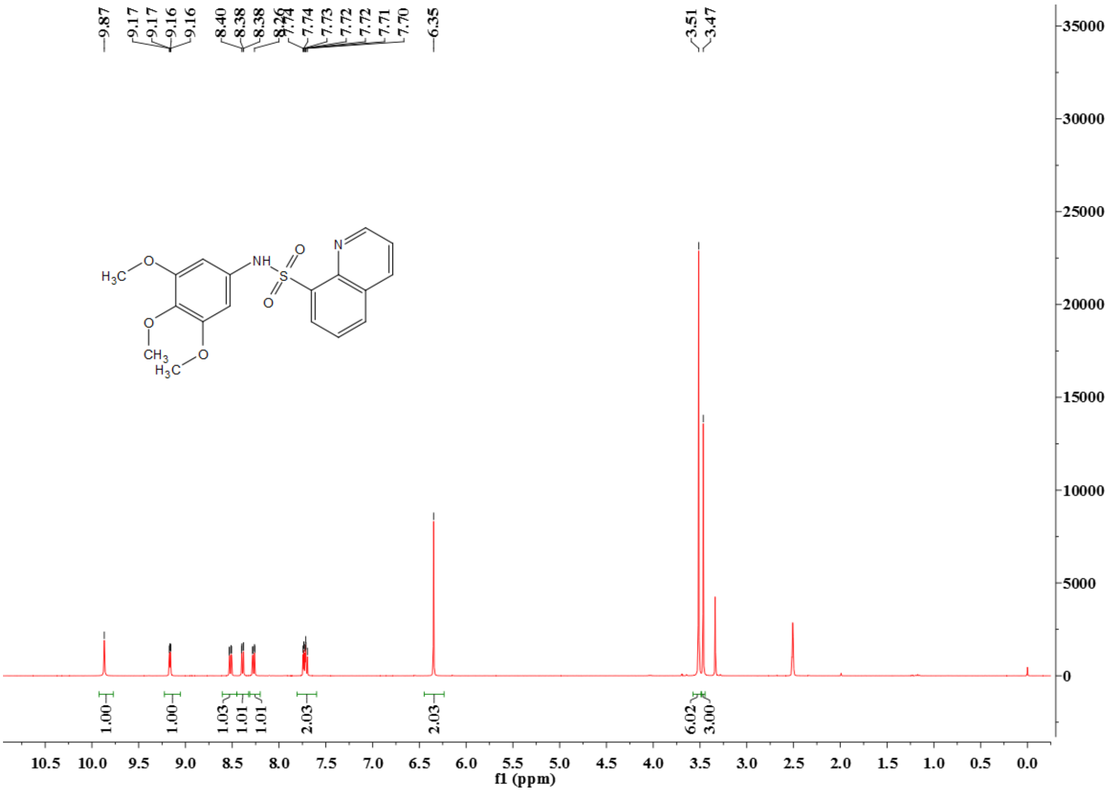

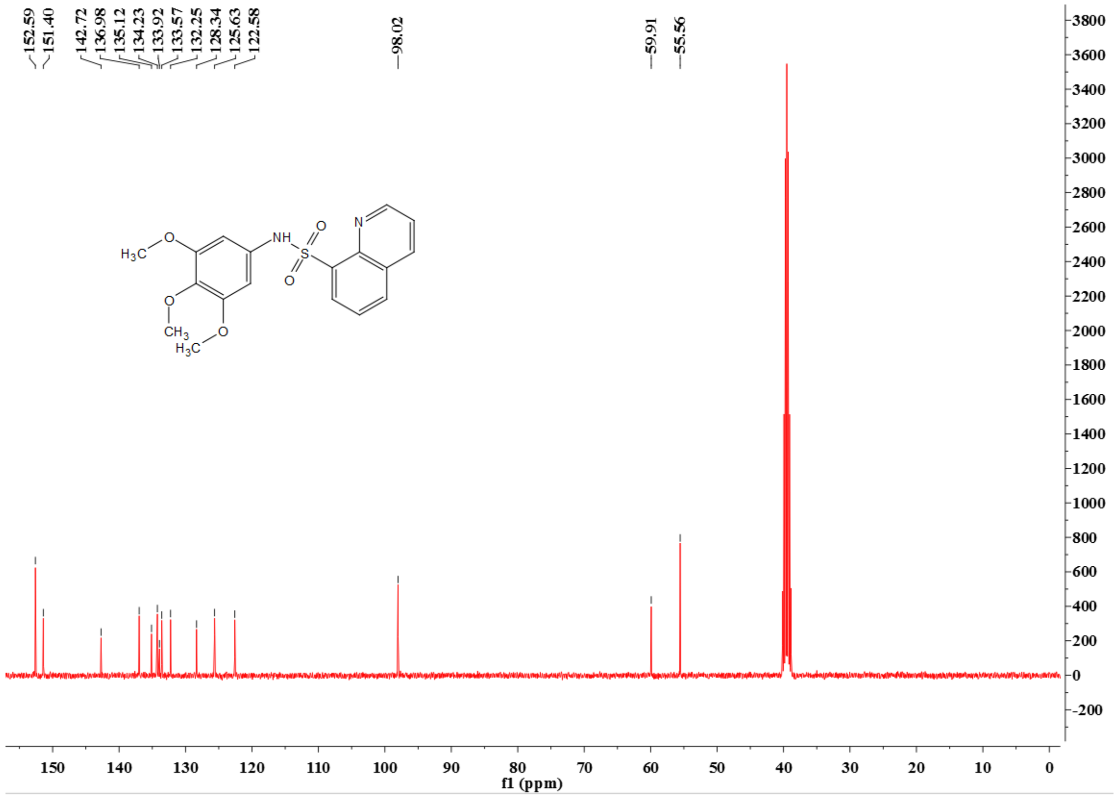


***N*-(3-bromopropyl)-*N*-(3,4,5-trimethoxyphenyl)quinoline-8-sulfonamide (12)**

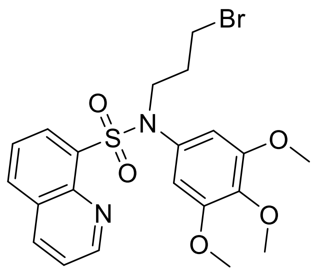

White solid, yield:82%, m.p.:129~131°C. $^{1}$H NMR (400 MHz, $CDCl_3$) δ 9.09 (dd, *J* = 4.2, 1.7 Hz, 1H), 8.20 (td, *J* = 8.6, 1.5 Hz, 2H), 7.93 (dd, *J* = 8.2, 1.2 Hz, 1H), 7.55 – 7.39 (m, 2H), 6.12 (s, 2H), 4.22 (t, *J* = 6.8 Hz, 2H), 3.67 (d, *J* = 4.6 Hz, 3H), 3.47 (t, *J* = 6.8 Hz, 2H), 3.42 (d, *J* = 8.3 Hz, 6H), 2.09 (p, *J* = 6.8 Hz, 2H). $^{13}$C NMR (100 MHz, $CDCl_3$) δ 152.0, 150.1, 143.2, 136.6, 135.8, 135.6, 133.6, 133.1, 132.5, 127.7, 124.6, 121.0, 105.2, 59.8, 54.9, 51.0, 31.8, 29.5. HRMS (m/z): Calcd. $C_{21}H_{24}BrN_2O_5S$, $[M+H]^+$m/z: 495.0589, found: 495.0593.

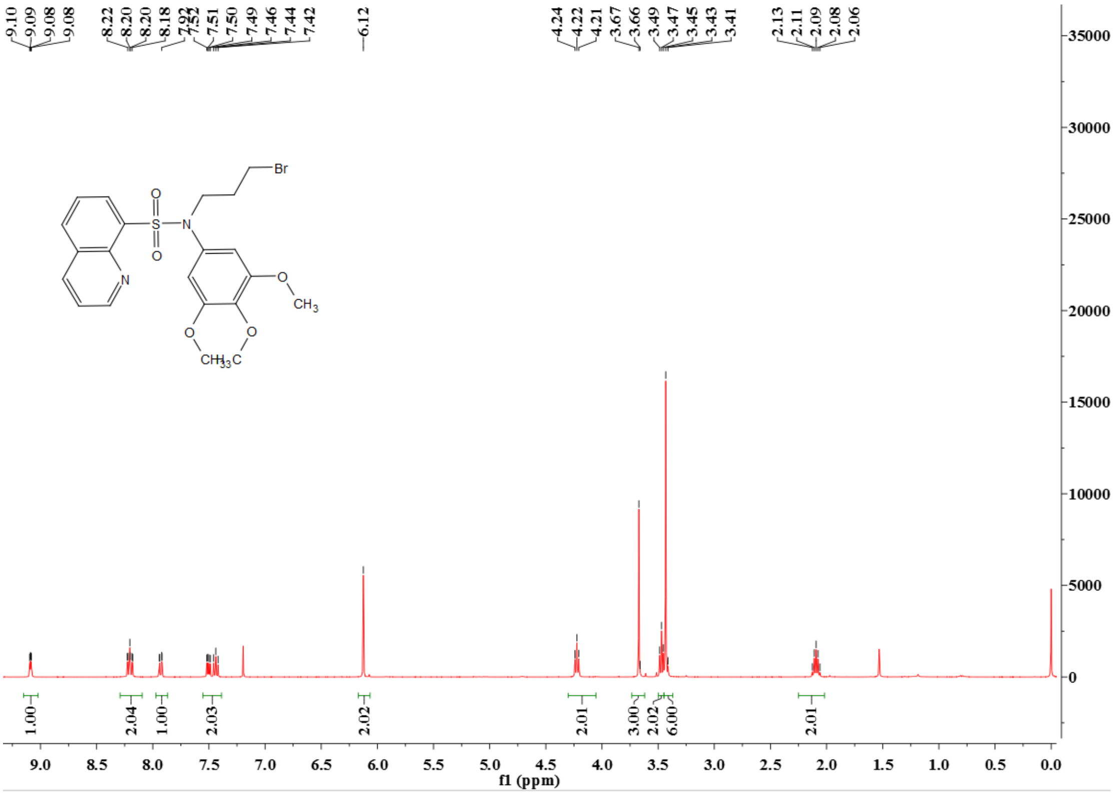


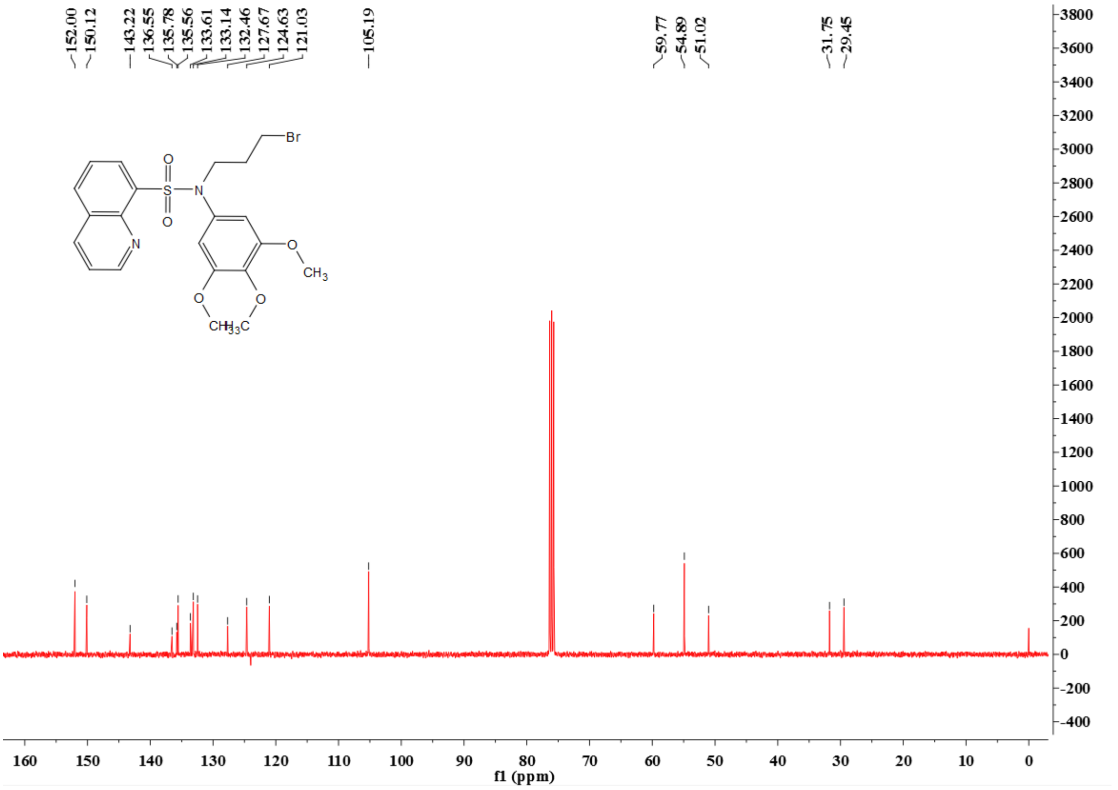


### *3-(N-(3,4,5-trimethoxyphenyl)quinoline-8-sulfonamido)propyl-4-methylpiperazine-1-carbodithioate (13a)*

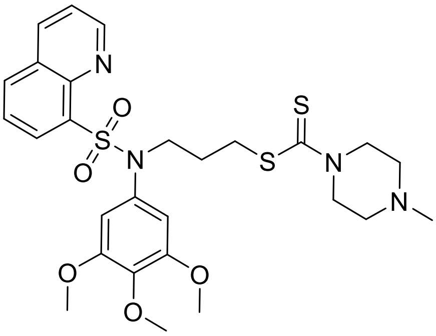


White solid, yield:76%, m.p.:96~98°C. $^{1}$H NMR (400 MHz, $CDCl_3$) δ 9.10 (dd, *J* = 4.2, 1.7 Hz, 1H), 8.37 – 8.08 (m, 2H), 7.93 (dd, *J* = 8.2, 1.2 Hz, 1H), 7.64 – 7.29 (m, 2H), 6.13 (s, 2H), 4.32 (s, 2H), 4.20 (t, *J* = 6.8 Hz, 2H), 3.96 (s, 2H), 3.67 (s, 3H), 3.42 (s, 6H), 3.40 (d, *J* = 7.3 Hz, 2H), 2.50 (s, 4H), 2.30 (s, 3H), 1.99 – 1.82 (m, 2H). $^{13}$C NMR (100 MHz, $CDCl_3$) δ 151.9, 150.2, 143.2, 136.5, 136.0, 135.5, 133.6, 133.1, 132.3, 127.7, 124.6, 121.0, 105.4, 59.8, 54.9, 53.2, 51.5, 44.3, 33.3, 27.8. HRMS (m/z): Calcd. $C_{27}H_{35}N_4O_5S_3$, $[M+H]^+$m/z: 591.1770, found: 591.1776.

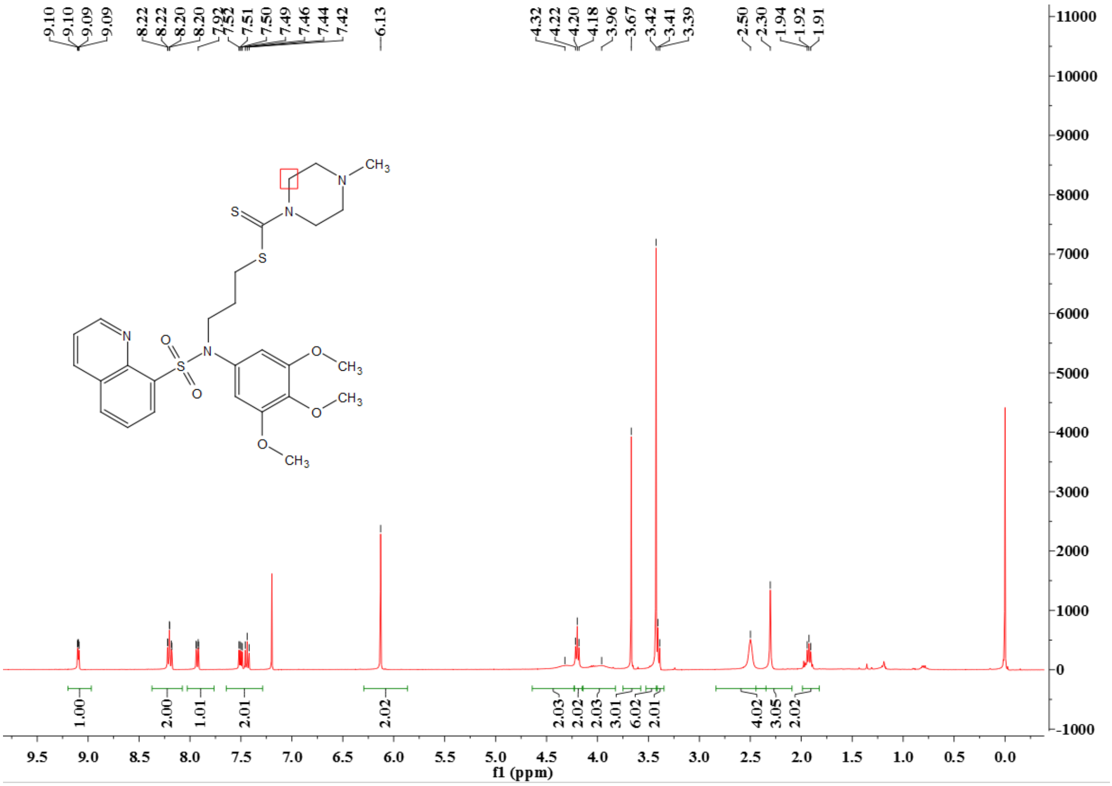

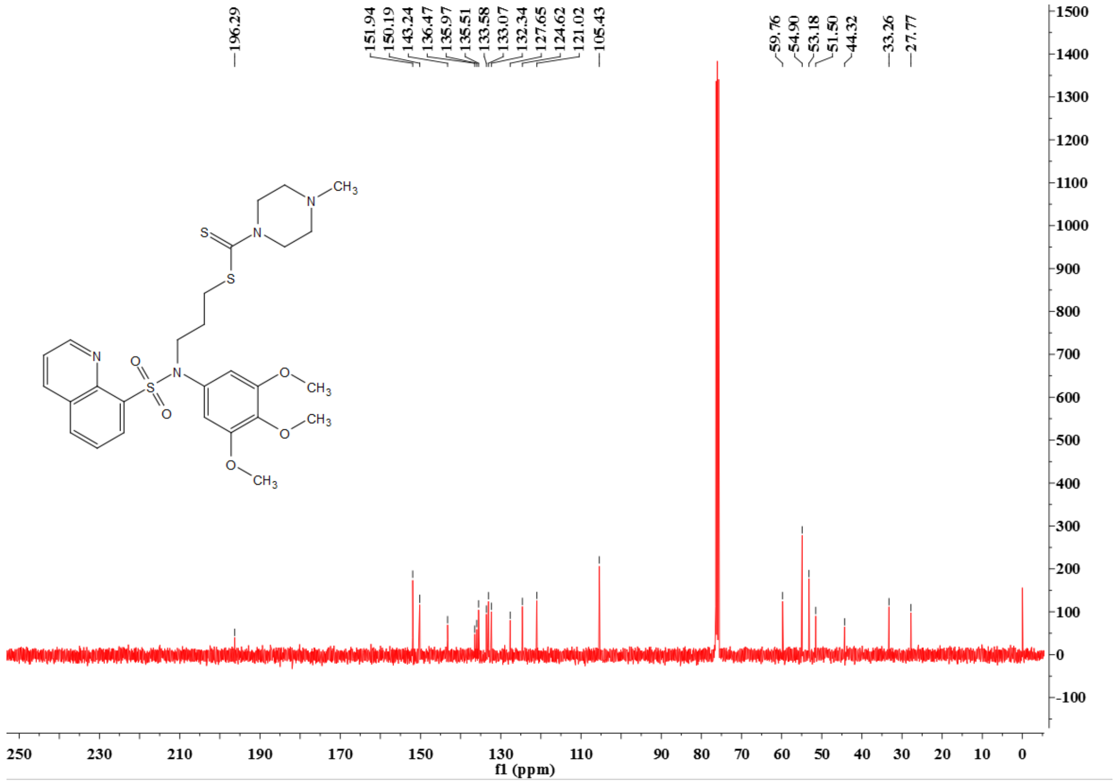


***3-(N-(3,4,5-trimethoxyphenyl)quinoline-8-sulfonamido)propyl-4-ethylpiperazine-1-carbodithioate(13b)***

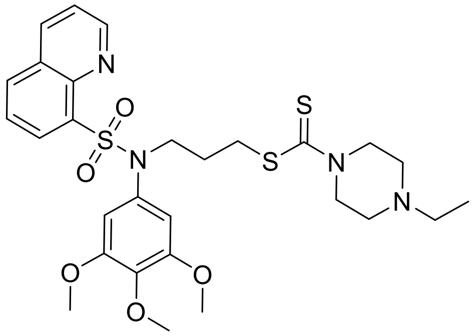

White solid, yield:84%, m.p.:131~133°C. $^{1}$H NMR (400 MHz, CDCl$_3$) δ 9.21 – 8.97 (m, 1H), 8.20 (dd, *J* = 9.8, 8.1 Hz, 2H), 7.92 (d, *J* = 7.7 Hz, 1H), 7.72 – 7.32 (m, 2H), 6.14 (s, 2H), 4.24 (d, *J* = 26.0 Hz, 2H), 4.20 (t, *J* = 6.7 Hz, 2H), 3.89 (s, 2H), 3.67 (s, 3H), 3.43 (s, 6H), 3.39 (d, *J* = 7.3 Hz, 2H), 2.64 – 2.17 (m, 6H), 1.99 – 1.83 (m, 2H), 1.03 (t, *J* = 7.2 Hz, 3H). $^{13}$C NMR (100 MHz, CDCl$_3$) δ 195.8, 151.9, 150.2, 143.3, 136.5, 136.0, 135.5, 133.6, 133.0, 132.3, 127.7, 124.6, 121.0, 105.5, 59.8, 54.9, 51.6, 51.2, 50.9, 33.1, 27.8, 11.0. HRMS (m/z): Calcd. $C_{28}H_{37}N_4O_5S_3$, $[M+H]^+$m/z: 605.1926, found: 605.1929.

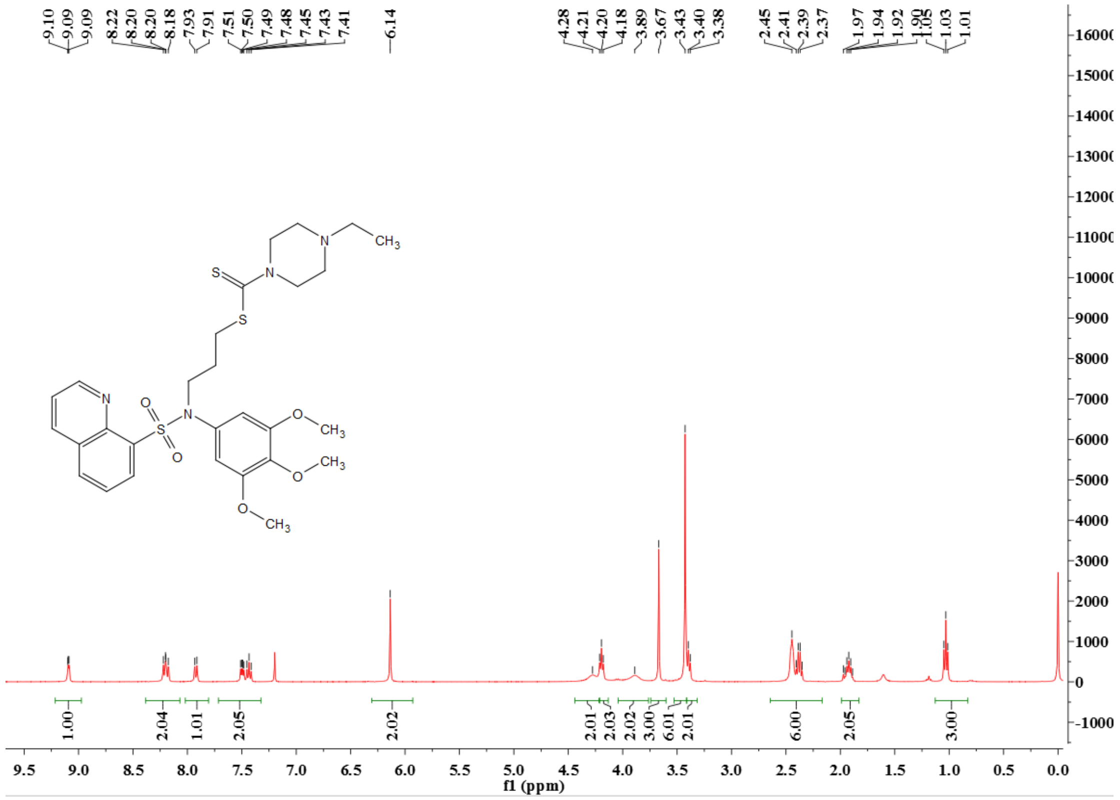


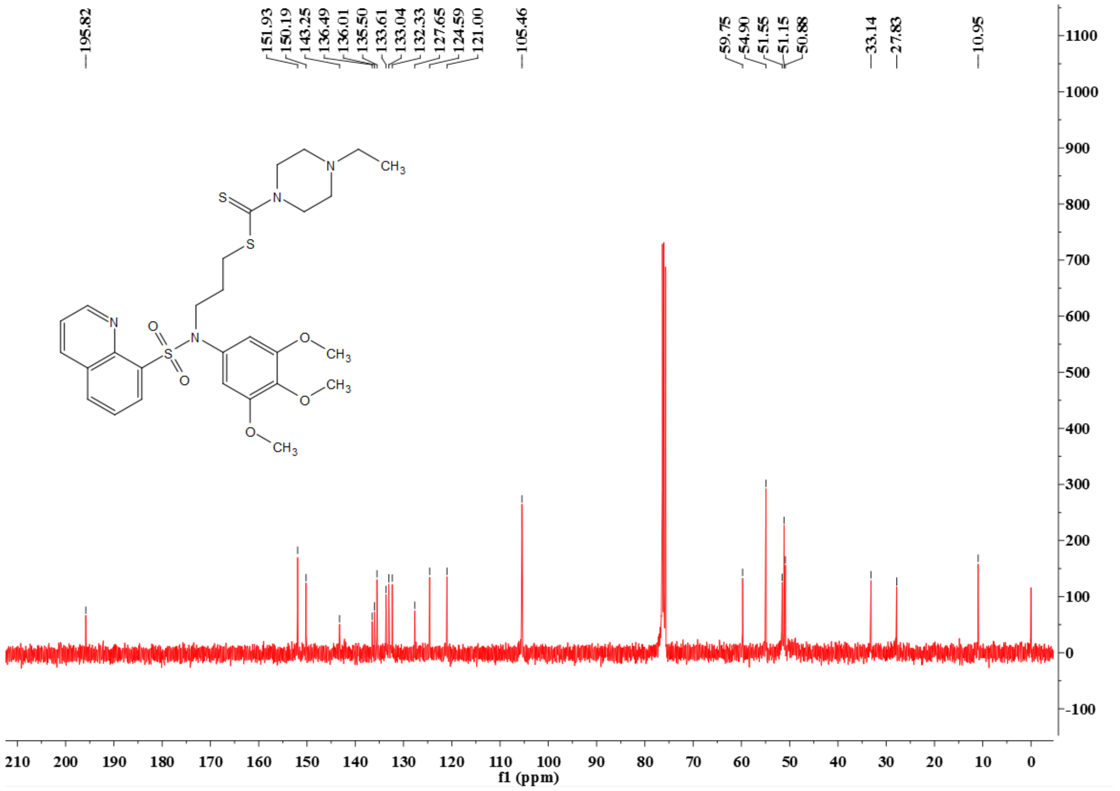


***3-(N-(3,4,5-trimethoxyphenyl)quinoline-8-sulfonamido)propyl-4-(2-hydroxyethyl)piperazine-1-carbodithioate(13c)***

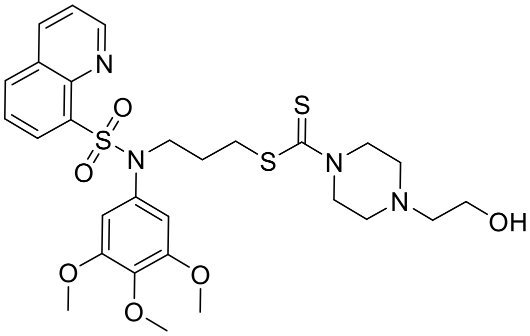


White solid, yield:75%, m.p.:106~108°C. $^{1}$H NMR (400 MHz, $CDCl_3$) δ 9.10 (d, *J* = 2.1 Hz, 1H), 8.20 (dd, *J* = 6.8, 3.9 Hz, 2H), 7.93 (d, *J* = 8.1 Hz, 1H), 7.65 – 7.34 (m, 2H), 6.12 (s, 2H), 4.33 (s, 2H), 4.20 (t, *J* = 6.6 Hz, 2H), 3.96 (s, 2H), 3.67 (s, 3H), 3.66 – 3.57 (m, 2H), 3.42 (s, 8H), 2.73 – 2.49 (m, 6H), 1.93 (dd, *J* = 13.7, 6.8 Hz, 2H). $^{13}$C NMR (100 MHz, $CDCl_3$) δ 196.3, 151.9, 150.2, 143.2, 136.3, 135.9, 135.5, 133.5, 133.1, 132.4, 127.6, 124.6, 121.0, 105.3, 59.8, 58.2, 56.7, 54.9, 51.5, 51.3, 33.2, 27.7. HRMS (m/z): Calcd. $C_{28}H_{37}N_4O_6S_3$, $[M+H]^+$m/z: 621.1875, found: 621.1878.

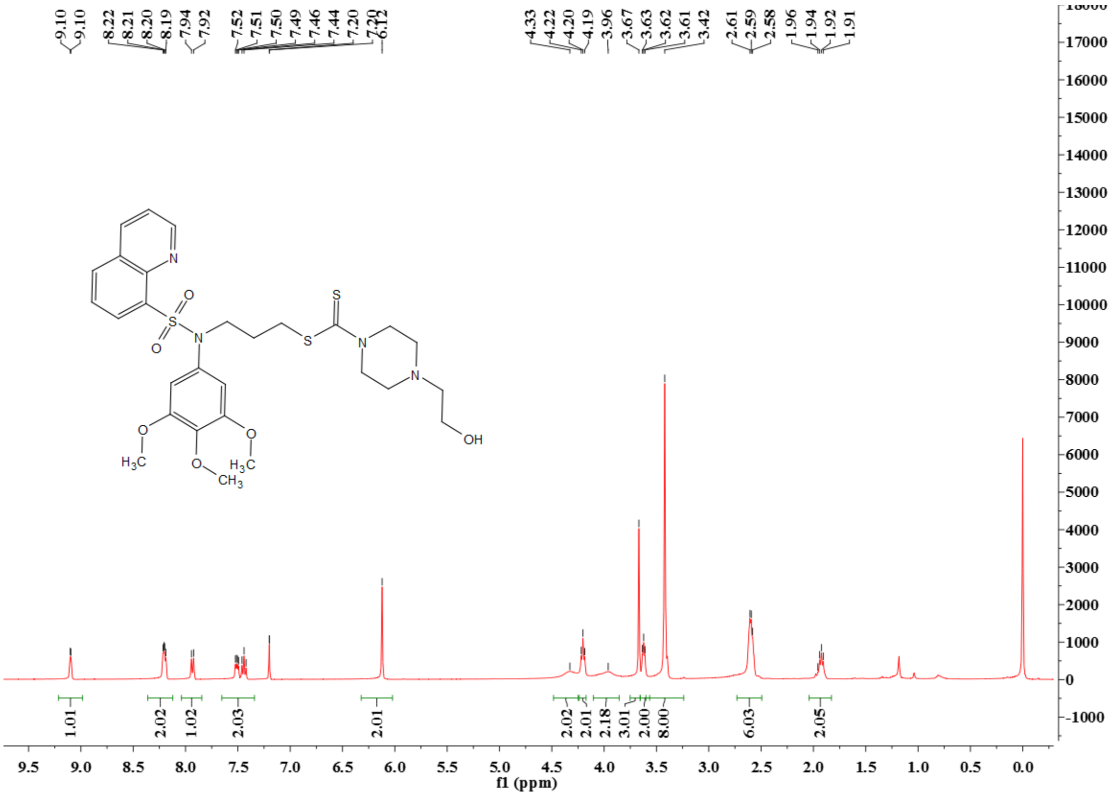

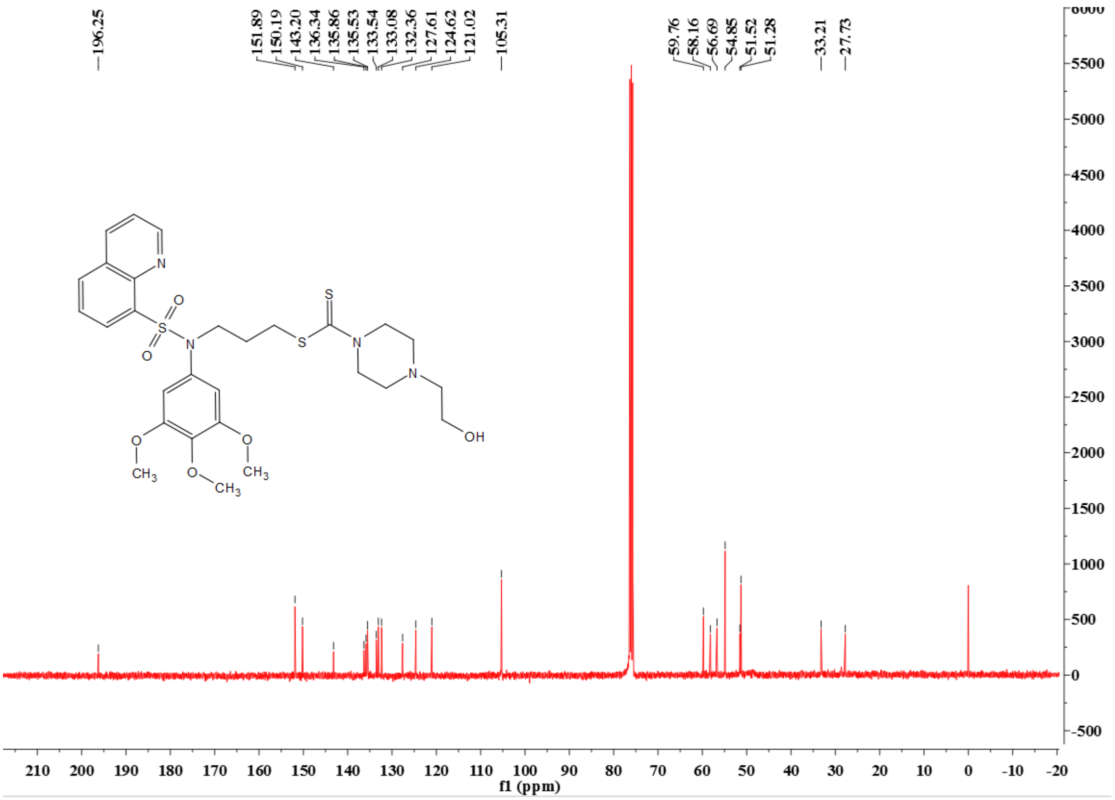

***3-(N-(3,4,5-trimethoxyphenyl)quinoline-8-sulfonamido)propyl-4-acetylpiperazine-1-carbodithioate (13d)***

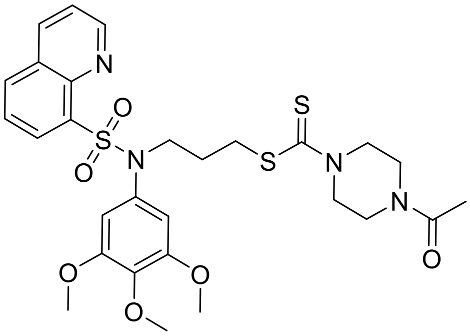

White solid, yield:90%, m.p.:128~129$^{o}$C. $^{1}$H NMR (400 MHz, $CDCl_3$) δ 9.09 (dd, *J* = 4.2, 1.7 Hz, 1H), 8.27 – 8.09 (m, 2H), 7.93 (dd, *J* = 8.2, 1.1 Hz, 1H), 7.56 – 7.33 (m, 2H), 6.13 (s, 2H), 4.21 (t, *J* = 6.7 Hz, 2H), 4.19 (s, 4H), 3.66 (s, 3H), 3.65 (s, 2H), 3.55 – 3.49 (m, 2H), 3.45 (s, 2H), 3.42 (s, 6H), 2.06 (s, 3H), 1.94 (dd, *J* = 14.9, 7.7 Hz, 2H). $^{13}$C NMR (100 MHz, $CDCl_3$) δ 197.0, 168.4, 152.0, 150.2, 143.2, 136.5, 135.9, 135.6, 133.5, 133.1, 132.4, 127.6, 124.6, 121.0, 105.4, 59.8, 54.9, 51.6, 44.2, 39.6, 33.2, 27.7, 20.4. HRMS (m/z): Calcd. $C_{28}H_{35}N_4O_6S_3$, $[M+H]^+$m/z: 619.1719, found: 619.1726.

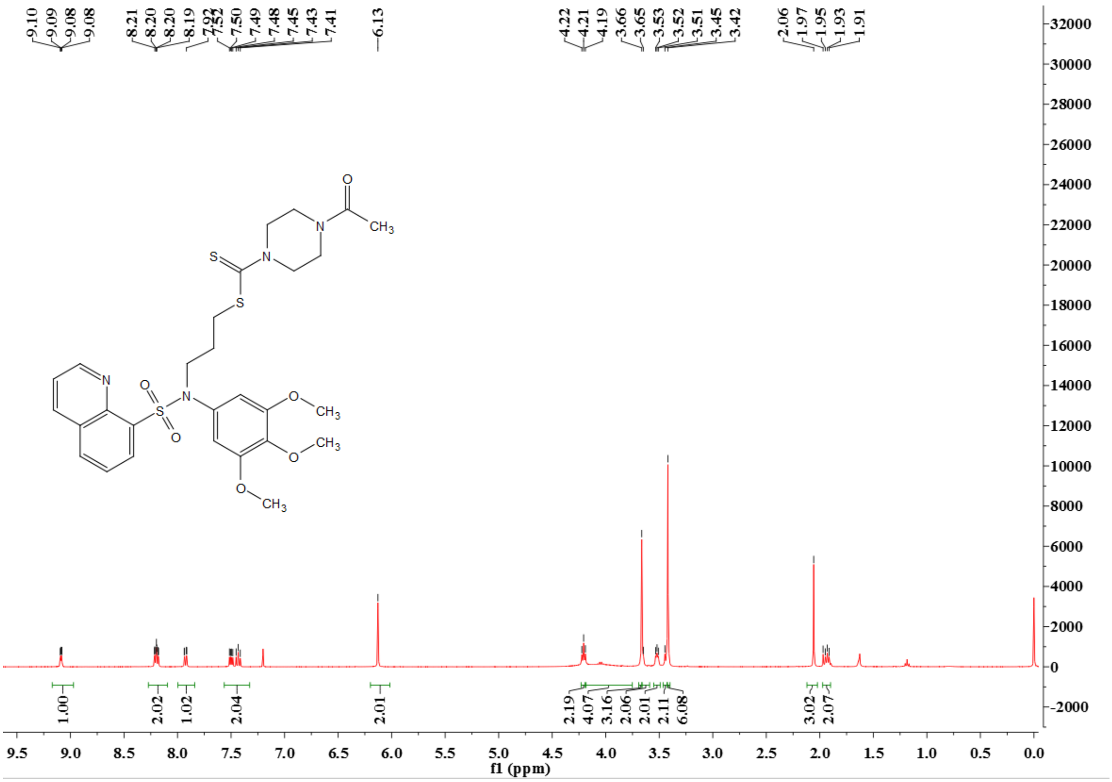
9.10 9.09 9.08 9.08 8.21 8.20 8.20 8.19 7.92 7.52 7.50 7.49 7.48 7.45 7.43 7.41
6.13
4.22 4.21 4.19 3.66 3.65 3.53 3.52 3.51 3.45 3.42
2.06 1.97 1.95 1.93 1.91
1.00 2.02 1.02 2.04 2.01 2.19 4.07 3.16 2.06 2.01 2.11 6.08 3.02 2.07
9.5 9.0 8.5 8.0 7.5 7.0 6.5 6.0 5.5 5.0 4.5 4.0 3.5 3.0 2.5 2.0 1.5 1.0 0.5 0.0
f1 (ppm)
32000 30000 28000 26000 24000 22000 20000 18000 16000 14000 12000 10000 8000 6000 4000 2000 0 -2000

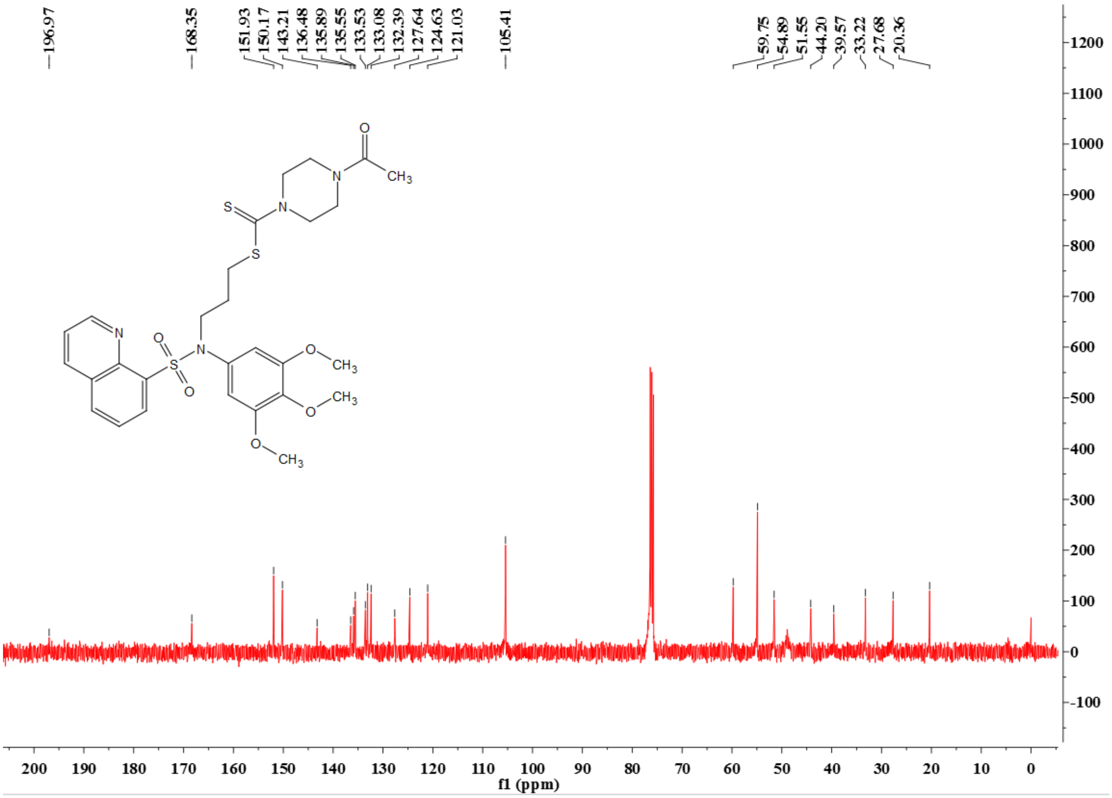
196.97
168.35
151.93 150.17 143.21 136.48 135.89 135.55 133.53 133.08 132.39 127.64 124.63 121.03
105.41
59.75 54.89 51.55 44.20 39.57 33.22 27.68 20.36
200 190 180 170 160 150 140 130 120 110 100 90 80 70 60 50 40 30 20 10 0
f1 (ppm)
1200 1100 1000 900 800 700 600 500 400 300 200 100 0 -100
